# Tendon release reduced joint stiffness with unaltered leg stiffness during gait in spastic diplegic cerebral palsy

**DOI:** 10.1371/journal.pone.0245616

**Published:** 2021-01-15

**Authors:** Chien-Chung Kuo, Hsing-Po Huang, Ting-Ming Wang, Shih-Wun Hong, Li-Wei Hung, Ken N. Kuo, Tung-Wu Lu

**Affiliations:** 1 Department of Orthopedics, China Medical University Hospital, Taiwan, R.O.C; 2 Department of Orthopedics, School of Medicine, China Medical University, Taiwan, R.O.C; 3 Department of Biomedical Engineering, National Taiwan University, Taiwan, R.O.C; 4 Department of Orthopaedic Surgery, School of Medicine, National Taiwan University, Taiwan, R.O.C; 5 Department of Orthopaedic Surgery, National Taiwan University Hospital, Taiwan, R.O.C; 6 Department of Physical Therapy, Tzu Chi University, Taiwan, R.O.C; University of Illinois at Urbana-Champaign, UNITED STATES

## Abstract

Biomechanical deviations at individual joints are often identified by gait analysis of patients with cerebral palsy (CP). Analysis of the control of joint and leg stiffness of the locomotor system during gait in children with spastic diplegic CP has been used to reveal their control strategy, but the differences between before and after surgery remain unknown. The current study aimed to bridge the gap by comparing the leg stiffness—both skeletal and muscular components—and associated joint stiffness during gait in 12 healthy controls and 12 children with spastic diplegic CP before and after tendon release surgery (TRS). Each subject walked at a self-selected pace on a 10-meter walkway while their kinematic and forceplate data were measured to calculate the stiffness-related variables during loading response, mid-stance, terminal stance, and pre-swing. The CP group altered the stiffness of the lower limb joints and decreased the demand on the muscular components while maintaining an unaltered leg stiffness during stance phase after the TRS. The TRS surgery improved the joint and leg stiffness control during gait, although residual deficits and associated deviations still remained. It is suggested that the stiffness-related variables be included in future clinical gait analysis for a more complete assessment of gait in children with CP.

## Introduction

Spastic diplegic cerebral palsy (CP) accounts for 22% of all types of cerebral palsy [1], with impaired motor control that may cause muscle tightness in the lower extremities, and postural and movement deviations [2]. Decisions regarding surgical treatment of spasticity are made based on the effects of neurological conditions on the bones and tissues of the growing child [3], aimed at maximizing function, reducing disability and facilitating mobility. Among the surgical procedures in treating spastic CP, such as osteotomy, muscle release and tendon transfer, tenotomy (tendon release) is often used to release the tendon to reduce the spasticity of the affected muscles for a greater passive range of motion while maintaining residual muscle tension [3–6]. Tenotomy is a simple, reliable and effective procedure with minor risks [5]. Previous studies have shown that tenotomy could significantly improve the range of motion in patients one year after surgery [4], reduce flexion deformity [7], and improve functional status and walking capabilities of these patients [8].

The efficacy of tendon release on CP during level walking has been predominantly evaluated using functional scores and/or clinical evaluation [9,10], such as using the Gross Motor Function Classification System (GMFCS) [11] and the Functional Mobility Scale (FMS) [12]. Computerized gait analysis has been used for identifying more detailed changes in walking performance in CP patients before and after tenotomy [13–17]. Depending on the location of the surgery, tenotomy was found to help one or more of the following: decreased hip flexion contracture, increased knee extension during stance, decreased knee flexion during swing and decreased ankle plantarflexion throughout the gait cycle [13–16]. Since the different joints and muscles of the lower extremities work together to meet the varying mechanical and balance demands during different phases of gait, changing the tightness of one muscle may affect the actions of the other muscles, and thus the motions of the joints. Identifying changes in individual muscles or joints without considering the coordination and interactions between muscles and joints may complicate the evaluation and interpretation of the gait data, and thus also of the CP gait performance [18]. A single index that combines the joint kinematics and kinetics of the lower extremities may be helpful for simplifying the evaluation of the overall performance of gait as an index for the efficacy of the surgical intervention.

Leg and joint stiffness during gait has been used for the assessment of pathology and of the efficacy of treatment because of its critical role in modulating the kinematics and kinetics of the locomotor system [19–21]. Changes in the leg and joint stiffness have been quantified in various motor tasks in different populations [19,21–25]. In these studies, the human body during walking was described as a simplified mass-spring model, with the leg modeled as a non-linear spring bearing the point mass of the whole body against collapse. With this model, the changes in the stiffness of the leg spring during gait represent the modulation of the kinematics and kinetics of the locomotor system via leg stiffness control. Following a similar approach, lower limb joints were modeled as a non-linear spiral spring, the stiffness changes of which represent the modulation of the joint angles and moments during gait [21,26]. The leg stiffness could be further decomposed into muscular (MC) and skeletal (SC) components according to DeVita and Hortobagyi [27]. The SC is related to the forces transmitted through the joints and bones, so a completely extended lower limb would have the leg stiffness provided solely by the SC. On the other hand, the muscular component is related to the muscular torques at the joints and the joint stiffness [26,27], so a flexing lower limb would be accompanied by an increasing MC but a decreasing SC. Their relative contributions to the leg stiffness can be described by the ratio of the MC and SC (MC/SC), which varies with the changes of the alignment of bones, and the muscle moments required to maintain the posture and movement during gait [21,27]. A previous study quantified the non-linear leg and joint stiffness changes during gait in children with spastic diplegic CP [21], and showed that leg stiffness played a critical role in modulating the kinematics and kinetics of the locomotor system. The children decreased their leg stiffness but increased the joint stiffness during most of the stance phase, indicating that they relied more on muscular contributions to achieve the required leg stiffness for maintaining body posture against collapse [21]. However, it remains unclear whether the leg stiffness (including skeletal and muscular components) and joint stiffness would be altered in response to a tenotomy in the lower leg in children with spastic diplegic CP.

The purpose of this study was to compare the leg and joint stiffness, the contributions of skeletal and muscular components, and the associated joint kinematics and kinetics in children with spastic diplegic CP during level walking before and after TRS *versus* healthy controls. It was hypothesized that, when compared to healthy controls, the CP group would have decreased leg stiffness but with increased muscular contributions and joints stiffness during gait, and that after TRS the CP group would increase the leg stiffness with reduced muscular contributions and joint stiffness when compared to those before TRS.

## Materials and methods

### Ethics statement

The work was approved by the Institutional Review Board of National Taiwan University Hospital, Taiwan, R.O.C (Permit Number: 201605138RIND). All participants were informed of the procedure and they provided written informed consent prior to the study. This observational, cohort study was conducted in the National Taiwan University Hospital Gait Laboratory.

### Subjects

A convenience sample of twelve children with spastic diplegic cerebral palsy (CP) (12 males; age: 10.9 ± 2.6 years; height: 140.7 ± 16.7 cm; mass: 32.6 ± 10.5 kg) was recruited from the outpatient orthopedic clinics at National Taiwan University Hospital during August 2016 to July 2019, and 12 healthy controls (12 males; age: 11.2 ± 2.3 years; height: 141.1 ± 12.6 cm; mass: 34.4 ± 9.3 kg) were recruited from the local community to match the patient group for sex, age, height and weight during the same time period (Table 1). Informed written consent signed by both the subjects and their legal guardians was obtained as approved by the Institutional Research Board. The subjects in the CP group met the following inclusion criteria: (1) graded I-III in the Gross Motor Function Classification System (GMFCS), (2) moderate to normal muscle strength, (3) mild to normal muscle tone, and (4) independent walking without assistive device. Participants with CP were excluded if they had pain, noticeable leg length discrepancies, serious muscle contractures, joint deformities, or any other pathology which might affect gait and/or cognitive function. The children with CP who participated in the current study were considered a good representation of typical children with GMFCS level II diplegic spastic CP in Taiwan. Each patient with CP received gastrocnemius and hamstring TRS performed by a senior paediatric orthopaedic surgeon (TMW) who made the decision based on clinical information and data from a standard clinical gait analysis on the patient. Distal gastrocnemius recession was performed by dividing the aponeurosis of the gastrocnemius. For the lengthening of the distal hamstrings, an open approach was used for the intramuscular aponeurotic lengthening of the semimembranosus, Z-lengthening of the semitendinosus, and either tenotomy or Z-lengthening of the gracilis at a level proximal to the knee. When the lateral hamstrings were included in the procedure, intramuscular aponeurotic lengthening of the biceps femoris was performed [28]. Before TRS, and six months to one year afterwards, each subject was evaluated for their lower limb stiffness control using computerized gait analysis. The healthy controls (Control) were free from any musculoskeletal, neurological or cardiovascular disorders. An *a priori* power analysis for a two-group independent sample *t*-test for the comparison of leg stiffness between diplegic CP and healthy children based on pilot results using GPOWER [29] determined that a projected sample size of seven subjects for each group would be needed with a power of 0.8 and a large effect size (Cohen’s *d* = 0.88) at a significance level of 0.05.

**Table 1:** Means (standard deviations) of the ranges of motion, muscle strength and muscle tone of the hip, knee and ankle joints for the diplegic CP group before (Pre-OP) and after (Post-OP) TRS. Relevant demographic details including: age, height, mass and GMFCS level for CP group and Controls.

		Range of Motion (degrees)	Muscle Strength (MMT)	Muscle Tone (Modified Ashworth Scale)
Hip				
Pre-OP				
Extension/Extensors		26.64 (6.57)	3.61(0.73)	0.15 (0.37)
Flexion/Flexors		116.8 (7.31)	3.95 (0.61)	0.15 (0.37)
Post-OP				
Extension/Extensors		23.21 (10.12)	3.95 (0.81)	0.08 (0.29)
Flexion/Flexors		116.9 (7.28)	4.42 (063)	0.04 (0.14)
Knee				
Pre-OP				
Extension/Extensors		-2.00 (6.63)	4.07 (0.53)	0.45 (0.70)
Flexion/Flexors		135.5 (2.13)	3.75 (0.48)	0.05 (0.16)
Post-OP				
Extension/Extensors		-1.42 (3.48)	4.42 (0.69)	0.31 (0.58)
Flexion/Flexors		134.5 (3.11)	4.10 (0.81)	0.00 (0.00)
Ankle				
Pre-OP				
Plantar-Flexion/Flexors		49.82 (5.00)	3.59 (0.84)	0.83 (0.64)
Dorsi-Flexion/Flexors		6.41 (10.88)	3.60 (0.57)	0.20 (0.42)
Post-OP				
Plantar-Flexion/Flexors		48.58 (6.64)	4.23 (0.61)	0.69 (0.83)
Dorsi-Flexion/Flexors		9.54 (9.44)	3.98 (0.65)	0.08 (0.29)
	Age (years)	Height (cm)	Mass (kg)	GMFCS
CP	10.9 (2.6)	140.7 (16.7)	32.6 (10.5)	II (0)
Controls	11.2 (2.3)	141.1 (12.6)	34.4 (9.3)	N/A

### Experimental protocol

In a hospital gait laboratory, each subject walked at a self-selected pace on a 10-meter walkway while the motions of the body segments were measured at 200 Hz via 39 retro-reflective markers placed on specific anatomical landmarks [30] using an 8-camera motion capture system (Vicon T-40s, OMG, U.K.), while the ground reaction forces (GRF) were measured at 2000 Hz using three forceplates (OR6-7-2000/Gen-5, AMTI, U.S.A.) [31,32]. Before data collection, the subjects were allowed to walk on the walkway several times to familiarize themselves with the experimental environment. Data from three gait cycles for each limb were obtained for subsequent analysis, both for the Control and for the CP group before and after TRS.

### Data analysis

The measured GRF and marker data were low-pass filtered using a fourth-order Butterworth filter with cut-off frequencies of 25 Hz and 10 Hz, respectively [33]. With the processed kinematic and GRF data, angular motions, and muscle moments at the lower limb joints were calculated using inverse dynamics analysis. Each body segment was modeled as a rigid body embedded with an orthogonal coordinate system with the positive x-axis directed anteriorly, the positive y-axis superiorly and the positive z-axis to the right in accordance with ISB recommendations [34]. A Cardanic rotation sequence of z-x-y was used to describe the rotational movements of each of the lower limb joints modeled as ball-and-socket joints [35]. Subject-specific body segmental inertial properties were obtained using an optimization-based method [36]. A global optimization method was used to reduce the effects of soft tissue artefacts associated with the skin markers on the pelvis-leg apparatus [37]. The calculated joint moments were normalized to body weight (BW) and leg length (LL), the latter defined as the length between the anterior superior iliac spine and the medial malleolus. Temporal-spatial parameters, namely walking speed, step length, stride time, cadence and step width were obtained. The stride length was calculated as the distance between the COP positions of two consecutive initial contacts of the same foot along the direction of progression, while the step width was calculated as the distance between two successive bilateral COP positions at initial contacts along the direction perpendicular to that of progression.

**Fig 1:**
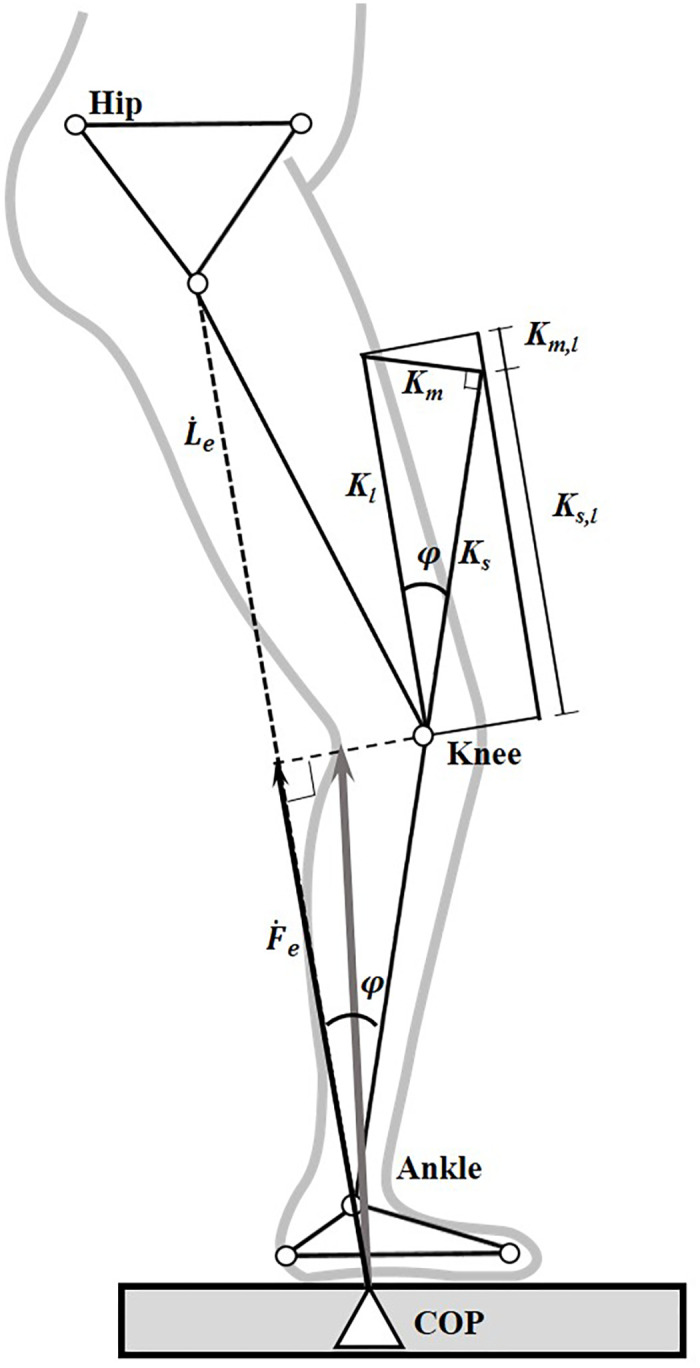
Model of leg stiffness, and skeletal and muscular components of the leg stiffness. Stick figure of a lower limb during stance phase of gait showing the definitions of the effective GRF (thin vector, Fe(t)) and effective leg length (Le(t)). φ(t) is the angle between the longitudinal axis of the shank and the line joining the COP of the GRF to the center of the hip joint; *K*_*s*,*l*_ (*t*) is the skeletal component in *K*_*l*_ (*t*); *K*_*m*,*l*_ (*t*) is the muscular component in *K*_*l*_ (*t*); *K*_*s*_(*t*) is the skeletal stiffness; and *K*_*m*_(*t*) is the muscular stiffness. (Adapted from [21]).

Each of the lower limbs was modeled as a non-linear spring which connected the center of pressure (COP) of the GRF and the hip joint center (Fig 1). The varying leg stiffness could thus be calculated as the slope (gradient) of the force vs. deformation curve during gait [21]. Similarly, the joint stiffness of the ankle, knee and hip were calculated as the slope of the moment vs. angle curve of the joint. The leg stiffness was further decomposed into muscular (MC) and skeletal (SC) components (Fig 1). The decomposition of the MC and SC was related to the angle (φ) between the longitudinal axis of the shank and the line joining the COP of the GRF to the center of the hip joint. While completely extended (φ = 0) joints of the lower limb would have the leg stiffness provided solely by the SC, the contribution of MC would increase with increasing φ [21,27] (Fig 1). The ratio of the MC and SC (MC/SC) was calculated to assess their relative contribution to the leg stiffness [21].

The leg stiffness, the skeletal and muscular components and their ratios, as well as the angles, moments and stiffness at the hip, knee and ankle in the sagittal plane, were calculated for each of the lower limbs. Time-averaged values of the variables were then calculated over the sub-phases of the stance phase of each limb, i.e., loading response (LR, initial double-limb support), mid-stance (MS), terminal stance (TS) and pre-swing (PS, terminal double-limb support). For each subject of both groups, the calculated variables from both limbs were averaged for subsequent statistical analysis.

### Statistical analysis

For each of the calculated variables, the time-averaged values of each sub-phase of the CP group were compared with those of the Control group for both Pre-OP and Post-OP using an independent *t*-test, while within-group comparisons of the CP group were performed using a paired *t*-test. All significance levels were set at α = 0.05. All the statistical analyses were performed using SPSS version 20.0 (SPSS Inc., Armonk, N.Y.: IBM, U.S.A.).

## Results

Compared to Control, both Pre-OP and Post-OP of the CP group showed significantly decreased gait speed and stride length but increased step width, while only the Pre-OP showed decreased cadence and increased stride time (p<0.05) (Table 2). No significant within-group differences in the temporal-spatial parameters were found in the diplegic CP group (p>0.05).

**Table 2:** Means (standard deviations) of the temporal-spatial parameters of gait for children with diplegic CP (Pre-OP: Before surgery; Post-OP: After surgery) and the Control group. P-values for pair-wise comparisons between Pre-OP, post-OP and Control are also given for each variable.

Group	Cadence (steps/min)	Gait speed (m/s)	Stride time (s)	Stride length (m)	Step width (m)
Control (CON)	119.37 (11.92)	1.08 (0.17)	1.02 (0.11)	1.08 (0.11)	0.10 (0.02)
CP Pre-OP (Pre)	107.60 (20.69)	0.60 (0.18)	1.14 (0.27)	0.68 (0.25)	0.17 (0.05)
CP Post-OP (Post)	100.18 (14.88)	0.57 (0.18)	1.24 (0.20)	0.70 (0.21)	0.18 (0.06)
*p* (CON vs. Pre)	0.106	<0.001*	0.161	<0.001*	<0.001*
*p* (CON vs. Post)	0.002*	<0.001*	0.002 *	<0.001*	0.001*
*p* (Pre vs. Post)	0.085	0.622	0.094	0.790	0.542

*p* (CON vs. Pre-OP): p-value between Pre-OP of diplegic CP group and Control.

*p* (CON vs. Post-OP): p-value between Post-OP of diplegic CP group and Control.

*p* (Pre-OP vs. Post-OP): p-value between Pre-OP and Post-OP of diplegic CP group.

*: significant difference (p<0.05).

**Fig 2:**
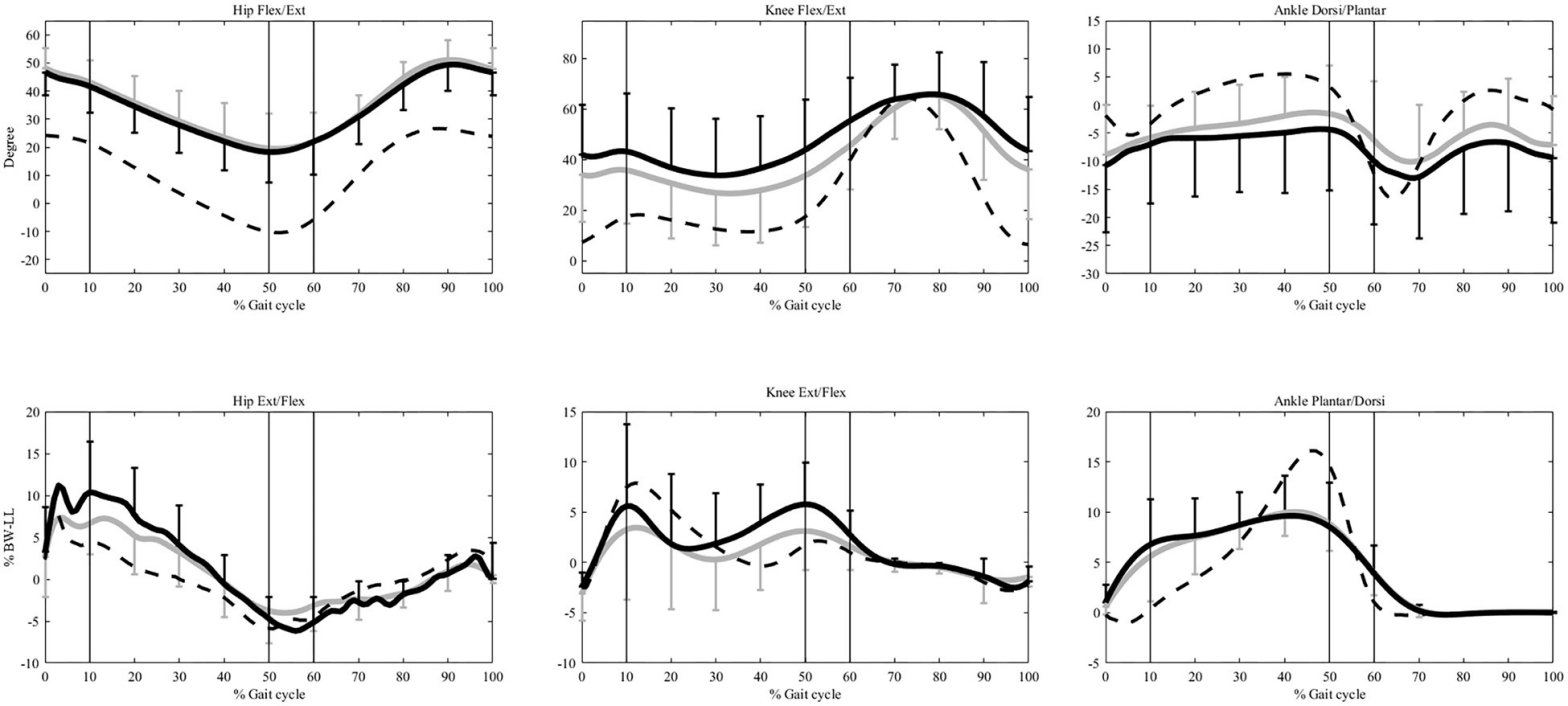
Ensemble-averaged joint angles and moments in the sagittal plane. The angles and moments at the hip, knee, and ankle for the control group are shown as dashed lines and those for the pre- and post-OP CP group as solid black and grey lines, respectively. The standard deviations are shown as one-sided error bars in each group. %BW*LL indicates the moments normalized to the body weight and leg length. The vertical lines from left to right are the beginning of single-limb support phase (contralateral toe-off), end of single-limb support phase (contralateral heel-strike), and the beginning of swing phase (toe-off), respectively.

Compared to Control, the CP group showed significantly increased hip and knee flexion in both Pre-OP and Post-OP throughout the stance phase, and increased plantar flexion during MS and TS, but increased ankle plantarflexion only in the Pre-OP during LR and PS (p<0.05) (Fig 2) (Table 3). The CP group showed increased hip extensor moments during MS to TS, increased ankle plantarflexor moments during LR to MS, but decreased ankle plantarflexor moments during TS to PS in both Pre-OP and Post-OP (Fig 2). The Pre-OP also showed increased hip extensor moments during LR, increased knee extensor moments during TS to PS, while the Post-OP showed a decreased hip flexor moment during PS, and a decreased knee extensor moment during MS when compared to the Control (p<0.05) (Fig 2) (Table 3). Compared to Pre-OP, the Post-OP of the CP group showed significantly decreased knee flexion angles during LR, TS and PS, decreased ankle plantarflexion angles during TS to PS, and significantly decreased hip extensor moments during LR, decreased hip flexor moments during PS, and decreased knee extensor moments during TS to PS (p<0.05) (Fig 2) (Table 3).

**Table 3:** Means (standard deviations) of the time-averaged angles and moments at the hip, knee and ankle in the sagittal plane over the stance phase of gait for children with diplegic CP and the Control group. P-values for pair-wise comparisons between Pre-OP, post-OP and Control are also given for each variable.

	Load Response (LR)	Mid-Stance (MS)	Terminal Stance (TS)	Pre-Swing (PS)
Hip Angle (degrees) (+/-: flexion/extension)				
Control (CON)	23.37 (6.05)	11.55 (4.30)	-3.73 (3.17)	-8.39 (4.88)
CP Pre-OP (Pre)	44.31 (8.03)	35.41 (9.16)	21.29 (9.00)	21.08 (9.16)
CP Post-OP (Post)	45.86 (6.93)	36.95 (8.60)	24.47 (11.66)	20.12 (12.00)
*p* (CON vs. Pre)	<0.001*	<0.001*	<0.001*	<0.001*
*p* (CON vs. Post)	<0.001*	<0.001*	<0.001*	<0.001*
*p* (Pre vs. Post)	0.484	0.519	0.631	0.956
Knee Angle (degrees) (+/-: flexion/extension)				
Control (CON)	13.76 (7.63)	16.16 (8.58)	12.70 (7.88)	25.21 (8.09)
CP Pre-OP (Pre)	42.31 (20.33)	38.01 (23.10)	37.04 (20.72)	48.65 (19.12)
CP Post-OP (Post)	35.23 (19.29)	32.26 (21.32)	28.52 (20.55)	37.90 (19.63)
*p* (CON vs. Pre)	<0.001*	0.006*	0.001*	0.001*
*p* (CON vs. Post)	0.002*	0.024*	0.021*	0.050*
*p* (Pre vs. Post)	0.031*	0.109	0.037*	0.027*
Ankle Angle (degrees) (+/-:dorsiflexion / plantarflexion)				
Control (CON)	-3.99 (3.06)	1.04 (4.73)	7.34 (3.17)	-0.11 (3.16)
CP Pre-OP (Pre)	-8.32 (4.94)	-4.97 (4.68)	-4.40 (5.60)	-7.69 (9.08)
CP Post-OP (Post)	-6.02 (5.62)	-4.19 (2.88)	-0.73 (5.55)	-1.38 (7.86)
*p* (CON vs. Pre)	0.030*	0.007*	<0.001*	0.017*
*p* (CON vs. Post)	0.325	0.006*	0.001*	0.625
*p* (Pre vs. Post)	0.367	0.290	0.049*	0.029*
Hip moment (%BW*LL) (+/-:extensor / flexor)				
Control (CON)	5.27 (1.50)	1.89 (0.87)	-2.42 (1.21)	-5.27 (1.16)
CP Pre-OP (Pre)	8.67 (5.42)	7.96 (5.31)	-0.57 (2.50)	-5.47 (2.58)
CP Post-OP (Post)	5.82 (2.56)	5.61 (3.25)	0.85 (2.35)	-3.12 (1.92)
*p* (CON vs. Pre)	0.048*	0.001*	0.032*	0.807
*p* (CON vs. Post)	0.525	0.001*	<0.001*	0.004*
*p* (Pre vs. Post)	0.035*	0.153	0.102	0.024*
Knee moment (%BW*LL) (+/-:extensor / flexor)				
Control (CON)	1.95 (1.99)	5.37 (2.07)	0.35 (0.38)	1.68 (0.57)
CP Pre-OP (Pre)	2.71 (2.88)	4.38 (3.80)	4.30 (3.39)	5.68 (3.29)
CP Post-OP (Post)	0.94 (3.76)	1.35 (5.49)	0.05 (2.32)	2.08 (2.62)
*p* (CON vs. Pre)	0.468	0.442	0.001*	0.001*
*p* (CON vs. Post)	0.416	0.028*	0.676	0.621
*p* (Pre vs. Post)	0.327	0.330	0.030*	0.025*
Ankle moment (%BW*LL) (+/-:plantarflexor / dorsiflexor)				
Control (CON)	- 0.95 (0.30)	2.41 (1.41)	12.23 (1.30)	8.97 (1.65)
CP Pre-OP (Pre)	4.25 (3.41)	7.63 (3.59)	9.34 (1.69)	6.23 (2.49)
CP Post-OP (Post)	2.45 (1.85)	6.99 (3.24)	9.47 (1.90)	6.71 (1.99)
*p* (CON vs. Pre)	<0.001*	<0.001*	<0.001*	0.005*
*p* (CON vs. Post)	<0.001*	<0.001*	<0.001*	0.007*
*p* (Pre vs. Post)	0.118	0.411	0.804	0.470

*p* (CON vs. Pre-OP): p-value between Pre-OP of diplegic CP group and Control.

*p* (CON vs. Post-OP): p-value between Post-OP of diplegic CP group and Control.

*p* (Pre-OP vs. Post-OP): p-value between Pre-OP and Post-OP of diplegic CP group.

*: significant difference (p<0.05).

BW: body weight; LL: leg length.

When compared to Control, both Pre-OP and Post-OP of the diplegic CP group showed significantly decreased leg stiffness during LR and PS with increased MC/SC ratio during LR and decreased SC during PS, while the Pre-OP also showed decreased leg stiffness during MS and an increased MC/SC ratio during MS to TS (p<0.05) (Table 4). Compared to the Pre-OP, the Post-OP maintained unaltered leg stiffness throughout the stance phase but showed a significantly decreased MC/SC ratio during most of the stance phase except during TS (Table 4).

**Table 4:** Means (standard deviations) of the leg and joint stiffness, and skeletal and muscular components of the leg stiffness in children with diplegic CP and the Control group during gait. P-values for pair-wise comparisons between Pre-OP, post-OP and Control are also given for each variable.

	Load Response (LR)	Mid-Stance (MS)	Terminal Stance (TS)	Pre-Swing (PS)
Leg Stiffness (N/m)				
Control (CON)	15.02 (6.42)	14.64 (5.98)	8.83 (2.02)	5.90 (2.25)
CP Pre-OP (Pre)	9.12 (5.63)	9.22 (4.09)	9.27 (3.60)	3.71 (1.60)
CP Post-OP (Post)	9.58 (4.70)	11.20 (5.87)	12.05 (5.98)	3.40 (1.89)
*p* (CON vs. Pre)	0.026*	0.017*	0.712	0.012*
*p* (CON vs. Post)	0.027*	0.169	0.091	0.008*
*p* (Pre vs. Post)	0.774	0.259	0.141	0.651
Skeletal Component (N/m)				
Control (CON)	14.69 (6.37)	13.87 (5.82)	8.01 (1.88)	4.60 (1.77)
CP Pre-OP (Pre)	7.82 (5.29)	8.11 (4.32)	7.69 (3.15)	2.65 (1.31)
CP Post-OP (Post)	8.74 (4.79)	10.36 (5.94)	10.89 (5.96)	2.67 (1.69)
*p* (CON vs. Pre)	0.009*	0.012*	0.764	0.006*
*p* (CON vs. Post)	0.017*	0.158	0.125	0.012*
*p* (Pre vs. Post)	0.569	0.203	0.084	0.974
Muscular Component (N/m)				
Control (CON)	0.33 (0.18)	0.76 (0.26)	0.81 (0.20)	1.30 (0.52)
CP Pre-OP (Pre)	1.35 (0.97)	1.14 (0.86)	1.50 (1.20)	1.04 (0.70)
CP Post-OP (Post)	0.85 (0.67)	0.85 (0.70)	1.25 (1.07)	0.71 (0.56)
*p* (CON vs. Pre)	0.002*	0.162	0.064	0.317
*p* (CON vs. Post)	0.016*	0.693	0.174	0.013*
*p* (Pre vs. Post)	0.058	0.047*	0.482	0.012*
Ratio of Muscular & Skeletal components (%)				
Control (CON)	2.59	6.18	10.40	30.29
CP Pre-OP (Pre)	21.77	19.91	21.97	47.81
CP Post-OP (Post)	13.66	12.54	15.25	33.02
*p* (CON vs. Pre)	0.001*	0.012*	0.027*	0.052
*p* (CON vs. Post)	0.016*	0.203	0.379	0.722
*p* (Pre vs. Post)	0.020*	0.013*	0.072	0.042*
Hip joint stiffness (%BW*LL /degree)				
Control (CON)	3.06 (1.00)	0.29 (0.06)	0.55 (0.16)	0.59 (0.19)
CP Pre-OP (Pre)	7.95 (5.92)	0.57 (0.32)	1.13 (0.70)	0.77 (0.38)
CP Post-OP (Post)	4.22 (2.98)	0.61 (0.60)	0.99 (0.36)	1.12 (0.50)
*p* (CON vs. Pre)	0.010*	0.007*	0.010*	0.157
*p* (CON vs. Post)	0.214	0.078	0.001*	0.003*
*p* (Pre vs. Post)	0.031*	0.833	0.544	0.097
Knee joint stiffness (%BW*LL /degree)				
Control (CON)	1.32 (0.36)	1.15 (0.23)	0.41 (0.13)	0.11 (0.03)
CP Pre-OP (Pre)	2.65 (1.79)	0.89 (0.21)	0.53 (0.29)	0.83 (0.59)
CP Post-OP (Post)	1.80 (0.75)	0.73 (0.26)	0.48 (0.16)	0.26 (0.21)
*p* (CON vs. Pre)	0.020*	0.009*	0.231	0.000*
*p* (CON vs. Post)	0.059	0.000*	0.260	0.021*
*p* (Pre vs. Post)	0.203	0.006*	0.696	0.011*
Ankle joint stiffness (%BW*LL /degree)				
Control (CON)	0.46 (0.13)	0.82 (0.30)	2.03 (1.17)	0.74 (0.18)
CP Pre-OP (Pre)	1.06 (0.53)	1.43 (0.84)	1.33 (0.80)	0.98 (0.65)
CP Post-OP (Post)	1.07 (0.69)	1.58 (0.79)	1.34 (0.50)	1.14 (0.61)
*p* (CON vs. Pre)	0.001*	0.026*	0.101	0.241
*p* (CON vs. Post)	0.006*	0.005*	0.073	0.043*
*p* (Pre vs. Post)	0.932	0.509	0.959	0.484

*p* (CON vs. Pre-OP): p-value between Pre-OP of diplegic CP group and Control.

*p* (CON vs. Post-OP): p-value between Post-OP of diplegic CP group and Control.

*p* (Pre-OP vs. Post-OP): p-value between Pre-OP and Post-OP of diplegic CP group.

*: significant difference (p<0.05).

BW: body weight; LL: leg length.

For the stiffness of individual joints, both Pre-OP and Post-OP of the CP group showed significantly increased joint stiffness at the hip during TS, at the knee during PS, at the ankle during LR to MS, and decreased joint stiffness at the knee during MS, when compared to Control. The Pre-OP also showed increased hip joint stiffness during LR to MS and increased knee joint stiffness during LR, while the Post-OP showed increased hip and ankle joint stiffness during PS (Table 4). Compared to Pre-OP, the Post-OP of the CP group showed decreased hip joint stiffness during LR, and decreased knee joint stiffness during MS and PS (p<0.05) (Table 4).

## Discussion

The current study aimed to quantify and compare the leg stiffness and the associated skeletal and muscular components, as well as lower limb joint stiffness during the stance phase of gait between healthy controls and children with diplegic CP before and after tendon release surgery. Before TRS, the patients with diplegic CP walked with decreased leg stiffness but with increased muscular contributions and joint stiffness when compared to healthy controls. After TRS, the CP group kept the leg stiffness unaltered with significantly decreased muscular contributions (i.e., decreased MS/SC ratio) and decreased joint stiffness at the hip and knee. This was accompanied with postural adjustments, including decreased knee flexion and decreased ankle plantarflexion during stance, for a more extended posture with reduced extensor moments at the knee and a more stable base of support (better foot contact).

The TRS appeared to improve the lower limb joint kinematics and kinetics of the diplegic CP group to be closer to those of the healthy controls, except for hip flexion and ankle plantarflexor moments which remained unchanged. The improvement included reduced hip extensor moments during LR, reduced knee flexion angles during most of the stance phase, and reduced knee extensor moments and ankle plantarflexion angles during TS and PS when compared to Pre-OP. These results suggest that release of the tight tendons of the gastrocnemius and hamstrings helped improve the motions at the ankle and knee, and thus the dynamic alignment of the lower limb segments, contributing to the reduction of the extensor moments at the knee and hip. The underlying strategy of such kinematic and kinetic changes could be revealed through further analysis of the stiffness-related variables.

During loading response, or during the initial double-limb support phase, the CP group showed significantly reduced leg stiffness with increased muscular contribution, as well as increased joint stiffness at the hip, knee and ankle before TRS when compared to the Controls. It has been suggested that the reduced leg stiffness in the CP group was as a result of the more flexed posture of the lower limbs during body weight-transfer, as was the increased effort of the muscles [21]. The TRS significantly decreased the hip joint stiffness and the muscular contributions to the leg stiffness to be closer to those of the Control while keeping the leg stiffness unaltered. The decreased joint stiffness at the hip helped reduce the effort for the subsequent extension with improved dynamic lower limb alignment for weight-transfer during this period. These changes may also be helpful for impact absorption with reduced muscle effort at heel-strike in the relatively jerky CP gait, reducing the energy expenditure and improving the endurance during the movement [38,39].

During mid-stance, similar to loading response, the CP group also showed significantly reduced leg stiffness with increased muscular contribution, as well as increased joint stiffness when compared to the Controls. These parameters are also related to the more flexed posture of the stance limb [21]. The TRS improved the lower limb alignment and maintained the leg stiffness with reduced, closer-to-normal knee joint stiffness and demands on the muscles. During terminal-stance, the CP group showed similar leg and joint stiffness values as compared to the Controls, except with increased muscular contribution and hip joint stiffness. Therefore, while the TRS improved the lower limb alignment, no significant changes on the leg and joint stiffness were found during this period. Overall, during single-limb support when the body was moving over the stationary foot, the TRS helped improve the lower limb alignment and achieve closer-to-normal muscular contributions to the leg stiffness, which will be helpful for reducing the risk of collapse owing to insufficient muscle strength when facing a sudden external load.

During pre-swing, or the terminal double-limb support, when the body weight was transferred to the contralateral limb, the CP group showed significantly reduced leg stiffness with reduced skeletal contribution, as well as increased knee joint stiffness when compared to the Controls. After the TRS, the leg stiffness was not altered but the knee joint stiffness and muscular contribution to the leg stiffness were significantly reduced, while the hip and ankle joints’ stiffness were increased. These changes showed the redistribution of the stiffness between the lower limb joints following TRS. The stiffer ankle and hip with a more flexible knee in the trailing limb were helpful for pushing the body forward and for the transfer of the body weight to the leading stance limb, as well as for the subsequent forward movement of the trailing limb. While the TRS helped improve the weight transfer performance during the pre-swing phase, it did not bring the leg and joint stiffness variables to normal values. Other surgical or rehabilitative intervention may be needed for further improvement of the remaining deviations in children with diplegic CP.

The current study showed that the TRS altered the stiffness control of the lower limb joints during walking while retaining an unaltered whole leg stiffness in children with diplegic CP. Such a change of control strategy was helpful for reducing the demand on the muscles in maintaining the same leg stiffness and body posture against collapse. However, residual deficits and associated deviations in leg and joint stiffness still remained. Rehabilitative training such as muscle strengthening after TRS may be needed for further improving the leg and joint stiffness control. The current study was the first attempt to use leg and joint stiffness to evaluate the efficacy of TRS in diplegic CP. The current patients were limited to GMFCS grade II; for children with different GMFCS grades, further studies will be needed. Another limitation was that the subjects in this study were all male patients. Further study would be needed to test whether the current results would apply to female patients. The current results encourage future application of such analysis in the assessment of the efficacy of orthopaedic and rehabilitative treatment in patients with walking impairments.

## Conclusions

The control strategy of body support during gait in children with diplegic CP after TRS was revealed through the analysis of leg and joint stiffness, the contributions of skeletal and muscular components, and the associated joint kinematics and kinetics. The CP group altered the stiffness of the lower limb joints and decreased the demand on the muscular components while maintaining an unaltered leg stiffness during stance phase after the TRS. The TRS surgery improved the joint and leg stiffness control during gait although residual deficits and associated deviations still remained. The analysis of leg stiffness and related variables in clinical gait analysis could provide more information on treatment effects in children with CP.

## Supporting information

S1 File (XLSX)
